# Heat tolerance around flowering in wheat identified as a key trait for increased yield potential in Europe under climate change

**DOI:** 10.1093/jxb/erv070

**Published:** 2015-03-07

**Authors:** Pierre Stratonovitch, Mikhail A. Semenov

**Affiliations:** Computational and Systems Biology Department, Rothamsted Research, Harpenden, Herts AL5 2JQ, UK

**Keywords:** Downscaling, heat stress, ideotype design, impact assessment, LARS-WG weather generator, Sirius wheat model.

## Abstract

To deliver food security for the 9 billon population in 2050, a 70% increase in world food supply will be required. Projected climatic and environmental changes emphasize the need for breeding strategies that delivers both a substantial increase in yield potential and resilience to extreme weather events such as heat waves, late frost, and drought. Heat stress around sensitive stages of wheat development has been identified as a possible threat to wheat production in Europe. However, no estimates have been made to assess yield losses due to increased frequency and magnitude of heat stress under climate change. Using existing experimental data, the Sirius wheat model was refined by incorporating the effects of extreme temperature during flowering and grain filling on accelerated leaf senescence, grain number, and grain weight. This allowed us, for the first time, to quantify yield losses resulting from heat stress under climate change. The model was used to optimize wheat ideotypes for CMIP5-based climate scenarios for 2050 at six sites in Europe with diverse climates. The yield potential for heat-tolerant ideotypes can be substantially increased in the future (e.g. by 80% at Seville, 100% at Debrecen) compared with the current cultivars by selecting an optimal combination of wheat traits, e.g. optimal phenology and extended duration of grain filling. However, at two sites, Seville and Debrecen, the grain yields of heat-sensitive ideotypes were substantially lower (by 54% and 16%) and more variable compared with heat-tolerant ideotypes, because the extended grain filling required for the increased yield potential was in conflict with episodes of high temperature during flowering and grain filling. Despite much earlier flowering at these sites, the risk of heat stress affecting yields of heat-sensitive ideotypes remained high. Therefore, heat tolerance in wheat is likely to become a key trait for increased yield potential and yield stability in southern Europe in the future.

## Highlight

In a modelling study to optimize wheat ideotypes under climate change, heat tolerance around flowering was identified as a key trait for high yields in southern Europe.

## Introduction

A substantial increase in world food supply of 70–100% is required to feed an estimated 9 billion people by 2050 (Godfray *et al.*, 2010). Because of limited possibilities to extend existing crop-growing areas, a considerable increase in crop productivity is needed to guarantee future food security (Parry *et al.*, 2011; Reynolds *et al.*, 2011). Wheat yields have increased significantly in the last century, mainly due to genetic improvements, higher fertilization rates and improved pest and diseases management (Semenov *et al.*, 2012). In the UK, for instance, average wheat yield has double since the 1960s, from 4 t ha^–1^ to 8 t ha^–1^. However, wheat crops are highly sensitive to environmental and climatic variations (Porter and Semenov, 2005). During the last two decades, the progress in wheat genetics has already been partly offset by changes in the European climate (Brisson *et al.*, 2010; Lobell *et al.*, 2011). Therefore, global warming, characterized by changing local weather patterns and increasing the frequency of extreme events, poses a major challenge to plant breeders in increasing yield potential (Semenov *et al.*, 2014). A multi-disciplinary approach is required to understand how plants respond to abiotic stresses and then to apply this knowledge in the context of climate change (Reynolds *et al.*, 2011; Zheng *et al.*, 2012; Martre *et al.*, 2015).

It has been shown that wheat is particularly sensitive to extreme cold and hot temperatures during the reproductive stage (Saini *et al.*, 1983; Marcellos and Single, 1984; Alghabari *et al.*, 2014; Vara Prasad and Djanaguiraman, 2014). With global warming, the frequency of high temperatures occurring around anthesis is predicted to increase in Europe (Semenov and Shewry, 2011). On the other hand, a modelling study showed that warmer temperatures in Australia can also increase the risk of frost damage in wheat crops by accelerating plant development so that anthesis coincides with late frosts (Fuller *et al.*, 2007). In order to quantify how extreme temperatures, low or high, could affect yield losses under climate change, wheat responses to frost and high temperature around anthesis and grain filling were incorporated into the Sirius crop simulation model (Jamieson *et al.*, 1998b; Semenov and Shewry, 2011; Semenov *et al.*, 2014). Sirius is a well-validated model and was able to simulate accurately wheat growth and grain yields in a wide range of environments, including Europe, USA, New Zealand, and Australia, and in experiments mimicking conditions of climate change, e.g. Free-Air Carbon dioxide Enrichment (FACE) experiments (Jamieson and Semenov, 2000; Ewert *et al.*, 2002; Martre *et al.*, 2006; Lawless *et al.*, 2008; He *et al.*, 2012; Asseng *et al.*, 2013).

The objectives of our study were to assess wheat yield potential and the impact of heat stress under climate change in Europe using the latest projections from the CMIP5 ensemble of global climate models (Stocker *et al.*, 2013). The refined Sirius model was used to select the optimal combination of traits of heat-tolerant (HT) or heat-sensitive (HS) wheat ideotypes at six European sites with diverse climates. Two experimental datasets were utilized to characterize HT and HS responses to high temperatures in wheat. First, in the Hot Serial Cereal (HSC) experiment, grain yields of the spring cultivar ‘Yecora Rojo’, which was sown at regular interval over 2 years, were severely affected by extreme low and high temperatures (Wall *et al.*, 2011; White *et al.*, 2011; Ottman *et al.*, 2012). This allowed us to characterize the effect of high temperature on accelerated leaf senescence during grain filling, affecting both the HT and HS ideotypes of our study. In the second independent experiment, the highly heat-sensitive cultivar ‘Chinese Spring’ (Qin *et al.*, 2008) was grown in a controlled environment and was subjected to various temperature treatments from booting to maturity (Vara Prasad and Djanaguiraman, 2014). Parameters derived in this experiment were used to describe the reduction of grain number and potential grain weight affecting only the HS ideotypes in our study.

The refined version of Sirius was used, with its incorporated responses to extreme temperatures, to optimize HT and HS ideotypes at each site. A wheat ideotype was defined as a set of eight cultivar parameters that control wheat phenology, canopy architecture, leaf senescence, responses to water stress, and root water uptake. By changing these parameters, wheat growth and development was changed in response to climatic and environmental variations and it was possible to select ideotypes with better performance under future climates and environments. The importance of tolerance to high temperature occurring during flowering and grain filling was assessed by comparing HT with HS ideotypes optimized at a site. Because parameters related to heat tolerance were not optimized, it can be anticipated that, at the southern sites, the phenology of HS ideotypes will be strongly influenced by the occurrence of high temperatures. On the other hand, HT ideotypes could extend the growing season without any yield penalties. This allowed yield losses resulting from heat stress effects to be quantified directly, for example, the reduction ingrain number and, indirectly, for example, the reduction in yield due to early flowering for HS ideotypes.

## Incorporation of extreme temperature responses in Sirius

### Accelerated leaf senescence in response to high temperatures

In wheat, high temperatures, greater than 34 °C, accelerate leaf senescence which has a significant impact on grain yield (Wardlaw and Moncur, 1995). Early senescence reduces the total amount of light intercepted by the crop by shortening the duration of grain filling (Porter and Gawith, 1999; Asseng *et al.*, 2011). An approach to model-accelerated leaf senescence was used, based on maximum canopy temperature, similar to Asseng *et al.* (2011).

In Sirius, the duration of leaf senescence is expressed in thermal time and linked to the rank of the leaf in the canopy, i.e. later emerged leaves have a longer senescence. Daily thermal time is calculated from 3-hourly canopy temperatures estimated by Sirius (Weir *et al.*, 1984). In Sirius 2010, a daily leaf thermal time increment Δ*Τ* (°C) was calculated as the average of the sum of the 3-hourly temperatures above a base temperature *T*_b_=0 °C. To account for the acceleration of leaf senescence caused by high temperature, the 3-hourlly temperatures *T*_i_ are multiplied by an accelerated leaf senescence factor $R_{i}^{\text{L}}$ (dimensionless):

$$\Delta T=\overset{8}{\underset{i=1}{\sum }}max\left(0,\left(R_{i}^{\text{L}}\times T_{\text{i}}-T_{\text{b}}\right)\right)/8$$

where $R_{i}^{\text{L}}$ increases linearly from 1 when *T*_i_ exceeds *T* ^L^, i.e.:

$$R_{i}^{\text{L}}=\text{1}+max(0,T_{\text{i}}-T^{\text{L}})\times S^{\text{L}}$$

where *S* ^L^ (°C^–1^) is the slope of the senescence acceleration per unit of canopy temperature above *T* ^L^. As in Sirius 2010, grain filling ends prematurely if the canopy is fully senesced.

### Impact of extreme temperatures during flowering and seed set

During meiosis, temperatures exceeding 30 °C are reported to cause abnormal development of both ovary and anthers which reduces floret fertility and, consequently, the number of developing grains (Saini *et al.*, 1983, 1984; Grant *et al.*, 2011). Then, at the beginning of grain filling, temperatures above 35 °C affect the development of the endosperm which limits maximum grain weight (Hawker and Jenner, 1993). These adverse effects of heat on grain number and weight have been incorporated into Sirius by modifying the calculation of the potential yield determinants: grain number and potential grain weight. As for accelerated leaf senescence, the reduction of the potential yield in Sirius is based on canopy temperature. In the absence of heat stress, the sink capacity of the grains (*Y*_pot_, g m^–2^) is set to be the product of the potential number of grains by the potential weight of an individual grain, i.e.:

$$Y_{\text{pot}}=DM_{\text{ear}}\times N_{\text{pot}}\times W_{\text{pot}}$$

where *DM*_ear_ (g m^–2^) is the dry mass accumulated in ears prior to anthesis, *N*_pot_ (grains g^–1^) is the maximum number of grain per unit of ear dry mass, and *W*_pot_ (g grain^–1^) is the potential weight of a single grain. In the absence of abiotic stress, the parameter values of *N*_pot_=122.4 grains g^–1^ and *W*_pot_=65mg are large enough to provide sufficient sink capacity to accommodate newly produced and translocated biomass. Therefore, in the absence of abiotic stress, grain yield will be determined by the source capacity of the crop (Sinclair and Jamieson 2006). The model changes described here limit the sink capacity in response to low and high temperatures occurring around anthesis and seed set. The timing of heat susceptibility for the reduction of grain number and potential grain weight were chosen from the results of a controlled-environment experiment from Prasad and Djanaguiraman (2014). The first period affecting the number of grain was defined from 10 d before anthesis to anthesis which coincides with meiosis and fertilization. The second period affecting the potential weight of grain was defined from 5–12 d after anthesis.

To account for the effect of high temperature on meiosis and fertilization, the number of fertile grain produced per unit of ear dry mass is reduced when the maximum canopy temperature $T_{\text{max}}^{\text{A}}$ (°C) during a period from 10 d before to anthesis exceeds a threshold temperature *T* ^N^ (°C). In this case, the heat reduction factor of fertile grain number ($R_{H}^{\text{N}}$, dimensionless) decreases linearly from 1 to 0 when $T_{\text{max}}^{\text{A}}$ exceeds *T* ^N^, i.e.:

$$R_{H}^{\text{N}}=ma\text{x}\left(0,min\left(\text{1},\text{1}-\left(T_{\text{max}}^{\text{A}}-T^{\text{N}}\right)\times S^{\text{N}}\right)\right)$$

where *S* ^N^ (°C^–1^) is the slope of the grain number reduction per unit of canopy temperature above *T* ^N^. The frost reduction factor of fertile grain number ($R_{F}^{\text{N}}$, dimensionless) decreases linearly from 1 to 0 if the minimum canopy temperature $T_{\text{min}}^{\text{A}}$ during a period from –3 to +3 d around anthesis is below a 0 °C threshold, i.e.:

$$R_{F}^{\text{N}}=max\left(0,min\left(\text{1},T_{\text{min}}^{\text{A}}+\text{1}\right)\right)$$

The actual number N (grains g^*–*1^) of grain per unit of ear dry mass is the product of the potential number of grain by the heat and frost reduction factors, i.e.:

$$N=N_{\text{pot}}\times R_{H}^{\text{N}}\times R_{F}^{\text{N}}$$

After the reduction of grain numbers at flowering, the potential weight of single grains could be limited by heat stress during endosperm development. The potential weight of each grain is reduced if the maximum canopy temperature $T_{\text{max}}^{\text{s}}$ (°C) occurring at the beginning of grain filling, i.e. a period of from 5–12 d after anthesis, exceeds a threshold temperature *T* ^W^ (°C). The maximum weight of a grain is reduced linearly from *W*_pot_ when $T_{\text{max}}^{\text{s}}$ exceeds *T* ^W^, i.e.:

$$W=W_{\text{pot}}\times max\left(0,min\left(\text{1},\text{1}-\left(T_{\text{max}}^{\text{s}}-T^{\text{W}}\right)\times S^{\text{W}}\right)\right)$$

where *W* (g grain^–1^) is the actual potential weight of a single grain limited by heat stress and *S* ^W^ (°C^–1^) is the slope of the potential weight reduction per unit of canopy temperature above *T* ^W^. Grain filling ends prematurely if the actual grain sink capacity *Y*_lim_=*DM*_ear_×*N*×*W* (g m^–2^) has been filled.

### Calibration and validation

**Fig. 1.**
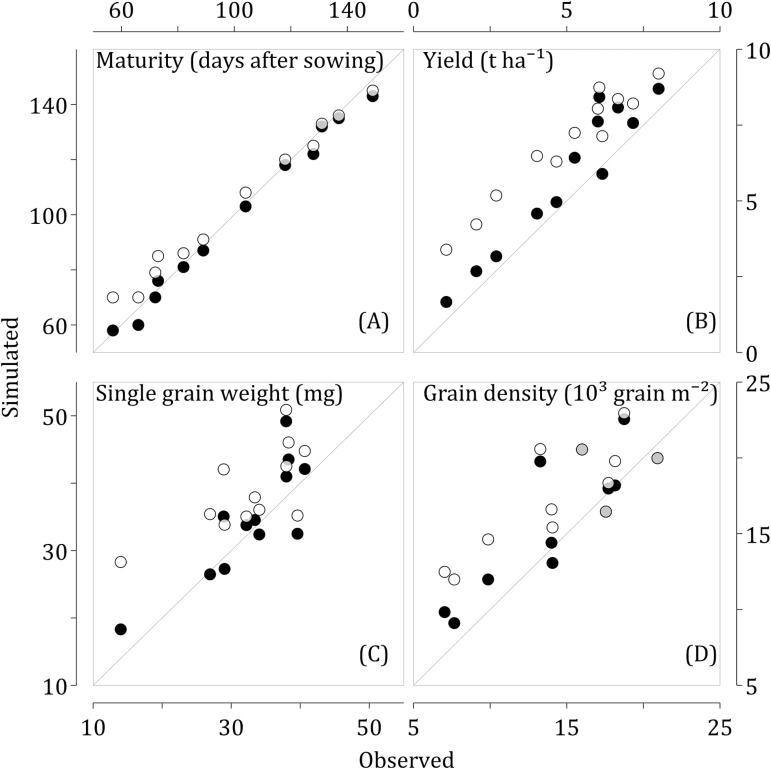
Simulated versus observed maturity dates (A), yield (B), single grain weight (C), and grain density (D) before (open circles) and after (closed circles) incorporation of high temperature responses in Sirius for the Hot Serial Cereal dataset. Points that overlap between both versions are shown in grey. The diagonals are the 1:1 lines.

The new parameters for responses to heat stress were calibrated using the Hot Serial Cereal (HSC) dataset (Wall *et al.*, 2011; White *et al.*, 2011; Ottman *et al.*, 2012). In this open field experiment, spring wheat was sown approximately every 6 weeks for 2 years in Maricopa, AZ. For six sowings, night/day temperatures were raised 1.3/2.7 °C in heated plots using infrared heaters. Before the incorporation of heat stress responses described above, Sirius was considerably overestimating yield, grain numbers, and grain weight for sowings where crops experienced very high temperatures from flowering to maturity. Furthermore, the difference between simulated and observed values increased with the increase of mean post-anthesis temperatures. After incorporation of the responses to high temperatures during anthesis and grain filling, the model errors were substantially reduced and no longer correlated with mean post-anthesis temperatures, as shown in Fig. 1.

## Ideotype design

The procedure to design a wheat ideotype for a changing climate was similar to that of Semenov *et al.* (2014). In brief, a wheat ideotype is characterized by eight Sirius cultivar parameters describing crop growth, development, and responses to abiotic stresses which are summarized in Table 1 and described in the section ‘Target ideotype traits’. An evolutionary algorithm was used to search for the optimal combinations of eight parameters that maximize yield under future climate for the selected sites and sowing dates presented in Table 2 (Semenov and Shewry, 2011). The performance of an ideotype is evaluated by computing 100 years mean yield for future climatic and environmental conditions. The future ideotypes are constrained to combinations of parameters with a coefficient of variation (CV) of yield lower than 15% and with a 90th percentile of the harvest index (HI) below a theoretical limit of 64% (Foulkes *et al.*, 2011; Semenov *et al.*, 2014). The constraint on CV allows for the selection of ideotypes with high yield stability.

**Table 1.** Sirius cultivar parameters with the value ranges used in ideotype optimization, from Semenov *et al.* (2014) (1) Using a model of canopy photosynthesis, it was shown that 10% in *L* could be achieved if λ (Rubisco specificity factor) was optimized (Zhu *et al.*, 2010). (2) Genetic variations of *Ph* up to 20% were observed for wheat (Mossad *et al.*, 1995; M. Ishag et al., 1998). (3) Varietal difference in number of days till heading under long and short day conditions varied between 9.74 and 107.40 in a photoperiodic response experiment (Košner and Žurková, 1996). (4) Genetic variation of *Gf* up to 40% was observed for wheat (Robert *et al.*, 2001; Charmet *et al.*, 2005; Akkaya *et al.*, 2006). (5) The reported range of genetic variation for flag leaf area under unlimited water and nitrogen was up to 40% (Fischer *et al.*, 1998; Shearman *et al.*, 2005). (6) Large genotypic variation in root characteristics and water uptake was reported (Asseng *et al.*, 1998; Manschadi *et al.*, 2006).

Parameter	Symbol	Range	
*Photosynthesis*			
Light conversion efficiency	*L*	1.10 (dimensionless)	(1)
*Phenology*			
Phyllochron	*Ph*	70–130 (°Cd)	(2)
Day length response	*Pp*	0.05–0.70 (leaf h^–1^ day length)	(3)
Duration of grain filling	*Gf*	500–900 (°Cd)	(4)
*Canopy*			
Maximum area of flag leaf	*A*	0.003–0.01 (m^2^ leaf m^–2^ soil)	(5)
‘Stay green’	*S*	0–1.5 (dimensionless)	
*Drought tolerance*			
Response of photosynthesis to water stress	*Wsa*	0.1–0.21 (dimensionless)	
Maximum acceleration of leaf senescence caused by water deficiency	*Wss*	1.2–1.9 (dimensionless)	
*Root water uptake*			
Rate of water uptake	*Ru*	1–7%	(6)

**Table 2.** Characteristics of six European sites and wheat cultivars (Semenov and Shewry, 2011) Annual precipitation and temperatures (°C) are from historical records (1980–2010). Abs. min.: absolute minimum of daily temperatures; Av. min.: monthly average of minimum temperature; Abs. max: absolute maximum of daily temperatures; Av. max: monthly average of maximum temperature.

Site	Country	ID	Longitude	Latitude	Annual precipitation	Temperature in January		Temperature in July		Cultivar	Sowing
					(mm)	Abs. min	Av. min	Abs. max	Av. max		
Tylstrup	Denmark	TR	9.9	57.2	704	–24.4	–2.3	31.2	20.3	Avalon	18/10
Rothamsted	UK	RR	–0.35	51.8	709	–11.1	1.2	33.8	21.7	Mercia	10/10
Debrecen	Hungary	DC	21.6	47.6	524	–28.3	–4.9	37.1	26.5	Thesee	18/10
Clermont-Ferrand	France	CF	3.1	45.8	583	–22.9	–0.3	40.7	25.6	Thesee	15/11
Montagnano	Italy	MO	11.8	43.3	690	–12.1	–1.0	39.3	31.1	Creso	25/11
Seville	Spain	SL	-5.88	37.42	572	–4.4	5.6	46.6	35.6	Cartaya	20/12

### Heat–sensitive and -tolerant ideotype

With the introduction of heat-stress responses in Sirius, the yield of HS ideotypes will be limited by high temperatures during flowering and grain filling (Table 3). In order to demonstrate the importance of tolerance to high temperature during the reproductive phase under climate change, ideotypes were optimized for future climatic conditions considering ideotypes to be either HS or HT. For a HS ideotype, the parameter values for grain number and potential grain weight reductions were derived from Prasad and Djanaguiraman (2014) with *T* ^N^=7 °C and *S* ^N^=0.125 °C^–1^, *T* ^W^=30 °C and *S* ^W^=0.004 °C^–1^. In their experiment, they selected the cultivar ‘Chinese Spring’ because of its known sensitivity to heat stress at flowering and at the beginning of grain filling (Qin *et al.*, 2008). The parameters for leaf senescence acceleration were set to *T* ^L^=28.9 °C and *S* ^L^=0.11 °C^–1^ after calibration using the HSC dataset (Asseng *et al.*, 2011; Ottman *et al.*, 2012). For a HT ideotype, grain number and maximum grain weight were not affected by high temperatures, i.e. *S* ^N^ and *S* ^W^ were set to 0. However, the acceleration of leaf senescence was the same as HS ideotypes. Both HS and HT ideotypes were sensitive to frost around anthesis. Other cultivar parameters of these ideotypes were set to the parameters values of the wheat cultivars selected for each site (Table 2).

**Table 3.** Parameters for Sirius responses to high temperature during anthesis and grain filling used for heat-sensitive (HS) and heat-tolerant (HT) ideotypes

Parameter	Symbol	HS	HT
*Leaf senescence*			
Temperature threshold (°C)	_*T*L_	28.93	
Slope of temperature increase (°C^–1^)	_*S*L_	0.108	
*Grain number*			
Temperature threshold (°C)	_*TN*_	27	NA
Slope of grain number reduction (°C^–1^)	_*S*N_	0.125	NA
*Maximum grain weight*			
Temperature threshold (°C)	_*T*W_	30	NA
Slope of maximum grain weight reduction (°C^–1^)	_*S*_^W^	0.004	NA

### Target environments

The six sites were selected to cover a range of contrasting wheat cropping environments in Europe from Denmark to Spain (Table 2). The climate projections from the HadGEM2-ES global climate model for a Representative Pathway Concentration (RCP) of 8.5 were used with LARS-WG weather generator to generate local-scale climate scenarios for each site for 2050 (Semenov and Stratonovitch, 2010). These scenarios contain 100 years of site-specific daily weather which were used to evaluate an ideotype performance during optimization. A single soil-water profile, Hafren, with a total available water capacity of 177mm, was used at all sites to eliminate site-specific soil effects from the analysis. Typical sowing dates for the baseline climate 1980–2010 and cultivars for each site are presented in Table 2 (Semenov and Shewry, 2011). The soil profile was filled to the maximum available water capacity at sowing. In all simulations, nitrogen was not limited. Following the RCP 8.5 scenario, the atmospheric CO_2_ concentration ([CO_2_]) was increased to 541 ppm compared with the baseline of 358 ppm. Radiation use efficiency is set to increase by 30% in Sirius for a doubling in [CO_2_], supported by recent field-scale experiments on the effect of [CO_2_] on C_3_ crops (Vanuytrecht *et al.*, 2012).

**Fig. 2.**
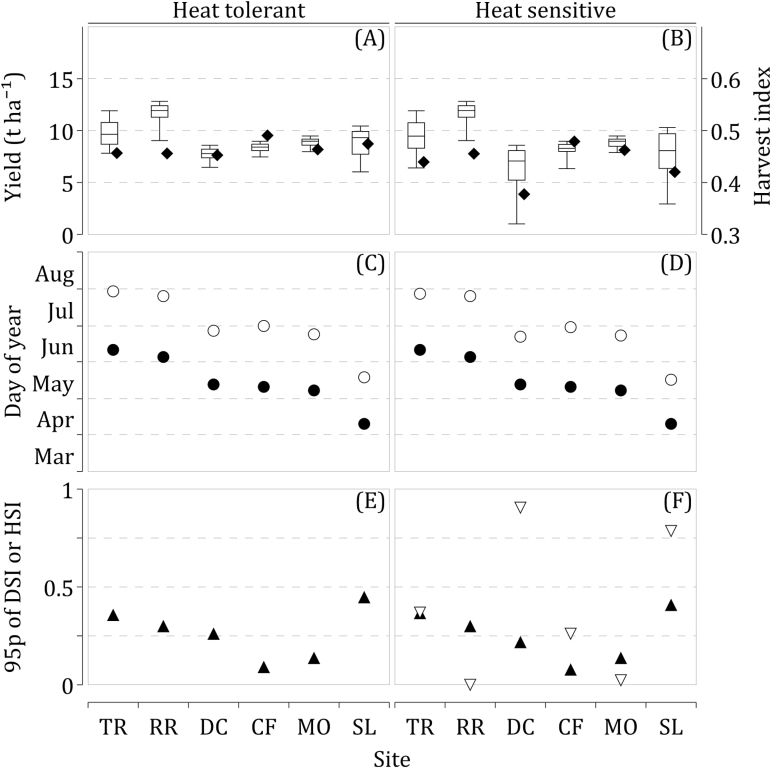
(A, B) Box plots of grain yield (whiskers: 5- and 95-percentiles; box: 25- and 75-percentiles; horizontal line: median) and harvest index (closed rhombuses), (C, D) mean anthesis (closed circles) and maturity (open circles) dates, and (E, F) 95-percentile of heat-stress index (HSI, open triangles) and drought-stress index (DSI, closed triangles) as simulated by Sirius using heat-tolerant (left) and heat-sensitive (right) current cultivars for the HadGEM2-ES (RCP8.5) climate scenario at six European sites. Information about sites, sowing dates, and chosen cultivars is given in Table 2.

To demonstrate the yield losses resulting from heat stress for a HS wheat cultivar, the Heat Stress Index was introduced, which is defined as the proportion of yield loss due to the effect of heat stress on yield, i.e. *HSI*=(*Y*_HT,WL_–*Y*_HS,ACT_)/*Y*_HT,WL_ where *Y*_HT,WL_ is the yield limited by water, but not heat stress, and *Y*_HS,ACT_ is the actual yield limited by water and heat stress as parameterized for ‘Chinese Spring’. This is similar to the Drought Stress Index calculated as *DSI*=(*W*_P_–*Y*_WL_)/*W*_P_ in Semenov *et al.* (2014) where *W*_P_ is the potential grain yield, i.e. not limited by water or heat stress. 95-percentile of HSI, HSI95, is a yield loss due to high temperatures occurring during the reproductive period, which could be expected, on average, once every 20 years. DSI95 is defined in a similar way. Figure 2 presents grain yields, harvest index, anthesis and maturity dates, as well as HSI95 and DSI95 predicted by Sirius for the current cultivars for future climate scenario, assuming that they were HT (left panels: A, C, E) or HS (right panels: B, D, F). Without considering the detrimental impact of high temperatures on fertilization and developing grains, wheat yields are predicted to increase in the future because CO_2_ fertilization offsets the shortening of the growing season caused by warmer temperatures (Fig. 2A, C). However, despite the reproduction period predicted to occur early in such conditions, the risk of high temperatures around anthesis and during grain filling remains high (Gouache *et al.*, 2012; Semenov *et al.*, 2014). Mean anthesis dates of HT and HS cultivars are identical by definition because they share the same phenological parameters (Fig. 2C, D). However, the mean maturity dates of HS cultivars were predicted to occur slightly earlier because of the reduction of the grain sink capacity caused by high temperatures on HS cultivars. On average, maturity of HS cultivars was reached 1.7 d earlier than HT cultivars, with the greatest difference found at Debrecen (DC; 5.2 d) and Seville (SL; 2.5 d). Figure 2E presents the DSI95 for the current cultivar assuming HT. Figure 2F presents DIS95 and HSI95 for the current cultivars if they were HS. At only two sites, RR (Rothamsted) and MO (Montagnano) is HSI95 negligible. At TR (Tylstrup), the most northern site of the study, HSI95 reaches 0.36. The loss expected at CF (Clermont-Ferrand) is lower at 0.26. The highest losses are predicted for DC and SL at 0.91 and 0.79, respectively. Consequently, future grain yields of the current cultivars with HS are projected to be lower and more variable compared with cultivars with HT at these four sites (Fig. 2B).

### Target ideotype traits

On top of the CO_2_ fertilization effect on radiation use efficiency described above, a 10% improvement in light conversion was assumed (Zhu *et al.*, 2008). General traits are explored here to understand how this increase in biomass accumulation could best be utilized given the future change in climate patterns. The parameters ranges selected for optimization were identical to Semenov *et al.* (2014) and are presented in Table 1. High-yielding traits were searched for both HT and HS ideotypes. For the HS ideotypes, the phenology parameters would be optimized to avoid high temperatures during the reproductive phase that substantially reduce grain yields.

#### Phenology

An optimal flowering time in relation to seasonal variations of solar radiation and water availability is a critical factor to maximize grain yield (Richards, 1991, 2006; Akkaya *et al.*, 2006). By modifying the phyllochron (*Ph*), i.e. the thermal time required for the appearance of successive leaves (Jamieson et al., 1995, 1998*a*, 2007), and the response of the final leaf number to day length (*Pp*) (Brooking *et al.*, 1995; Jamieson *et al.*, 1998*b*), the rate of crop development and, consequently, the timing of anthesis and maturity, are altered (Semenov *et al.*, 2014). The duration of grain filling (*Gf*) is also selected to avoid terminal drought and heat stress. Longer periods of grain filling increase the amount of radiation intercepted by the crop, potentially increasing yield (Evans and Fischer, 1999). However, because the rate of biomass relocation from the plant’s reserves in Sirius is inversely proportional to the duration of grain filling, a part of this resource might not be remobilized to the grain in the advent of terminal stress (Semenov *et al.*, 2014).

#### Canopy

The rate of canopy expansion and the maximum leaf area index is a key factor affecting the cumulative amount of radiation intercepted during the growing season; it also affects the transpiration demand from the plant. The potential size of the flag leaf layer (*A*) was selected to find an optimum canopy development with respect to biomass accumulation and transpiration demand (Semenov *et al.*, 2014). Delaying leaf senescence after anthesis is a possible strategy to increase grain yield because of the associated increased in biomass accumulation (Austin, 1999; Silva *et al.*, 2001; Triboi and Triboi-Blondel, 2002). A “stay-green’ parameter (*S*) was therefore included in optimization.

#### Tolerance to drought

In Sirius, daily photosynthesis and the rate of leaf senescence are both dependent on the ratio of actual to potential evapotranspiration. Tolerance of biomass assimilation (*Wsa*) and leaf senescence (*Wss*) to water stress are potentially important for southern Europe sites (Semenov *et al.*, 2014).

#### Root water uptake

The proportion of daily water extractable by the plant declines from 10% at the top of the soil to *Ru* at maximum root length. A slower rate of uptake might increase yield in drier environment by conserving water for the end of the cropping season (Manschadi *et al.*, 2006).

## Ideotype selection

A typical sowing date and a wheat cultivar were selected for each of the six sites (Table 2). For each site, 16 HT and 16 HS candidate ideotypes were designed independently by optimizing the eight parameters presented in Table 1. The design of first HT and first HS candidate ideotypes started with the eight parameter values of the cultivar selected for the site, while the remaining 15 HT and 15 HS designs started with the eight parameter values randomly selected within the parameters’ limits. The other ideotype parameters were set to the values of the cultivar chosen for the site. The optimization procedure was repeated at each site with the sowing date set 4 weeks earlier than those in Table 2 (a potential adaptation option). In total, 384, i.e. 6 sites×2 sowing dates×(16 HT+16 HS), candidate ideotypes were generated. For a single site and sowing date, the 16 HT and 16 HS candidate ideotypes converged to different sets of parameters in the search space because of the stochastic nature of the evolutionary algorithm used, the different starting positions, and the presence of local optimums. Therefore, at each site and for both sowing dates, representative HT and HS ideotypes were chosen from the 5 HT and the 5 HS candidates with the highest mean grain yield. For HT ideotypes, the mean grain yield achieved by the five best candidates were all very close, with only a 0.3 t ha^–1^ difference between the highest and the lowest yielding candidate at any one site. The candidate with the lowest DSI95 was selected in each site and for each sowing date as the representative HT ideotype. With the exception of the candidates generated for the current sowing date at SL, the mean grain yields of the five best HS candidates were also similar to each other, with the difference between the highest and lowest yield candidate being 0.6 t ha^–1^. At SL, the two highest yielding candidates for the current sowing date had a mean yield of 9 t ha^–1^ and 8.7 t ha^–1^, respectively. However, their CV of grain yield was around 30%, well above the 15% limit imposed during the ideotype design. This indicated that for these two parents (one of them was the initial cultivar) the search algorithm could not find combinations of parameters that reduced yield variability and the optimization stopped prematurely. The mean yield of the remaining three candidates was lower, 7.5 t ha^–1^ on average, and closer to each other. The candidate with the lowest sum of DSI95 and HSI95 was selected as the representative HS ideotype for a site and sowing date. Selected ideotypes were among the HS candidates with the highest yield stability and were better adapted to limit the severity of drought and heat stress.

## Impact of extreme temperatures on future ideotypes

**Fig. 3.**
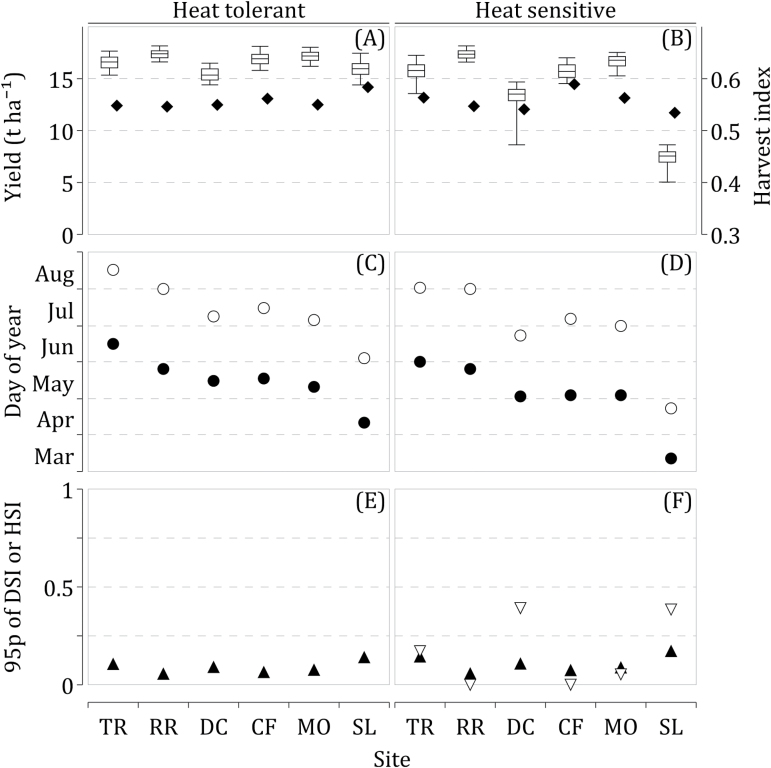
(A, B) Box plots of grain yield (whiskers: 5- and 95-percentiles; box: 25- and 75-percentiles; horizontal line: median) and harvest index (closed rhombuses), (C, D) mean anthesis (closed circles) and maturity (open circles) dates (changes from current cultivars presented as error bars), (E, F) 95-percentile of heat-stress index (HSI, open triangles) and drought-stress index (DSI, closed triangles) as simulated by Sirius using heat-tolerant (left) and heat-sensitive (right) ideotypes optimized for the HadGEM2-ES (RCP8.5) climate scenario at six European sites. Information about sites and sowing dates is given in Table 2.

Figure 3 presents grain yield, harvest index, anthesis and maturity dates, as well as HSI95 and DSI95 predicted by Sirius for selected HT (left panels: A, C, E) and HS (right panels: B, D, F) ideotypes. Mean anthesis and maturity dates, as well as relative change from current cultivars, are given in Table 4. A substantial yield increase was found for HT ideotypes compared with current cultivars for the HadGEM2-ES RCP8.5 scenario (Fig. 3A). This could be explained by an extended growing season (Table 4), optimized phenology, quick canopy establishment, and delayed leaf senescence (Fig. 3B) (Semenov *et al.*, 2014). Grain yields of ideotypes were higher than current cultivars by 83% on average, with the biggest increases at DC (+100%) and CF (+103%). On average, anthesis and maturity dates were brought 1.3 d and 13.4 d later, respectively (Fig. 3C). This extended the grain-filling period by an average of 12.1 d thereby increasing the amount of solar radiation intercepted by the crops. This delivered a high increase in yields because of the CO_2_ fertilization effect and the direct allocation of the biomass assimilated post-anthesis to the grains in Sirius. As a result, the mean harvest index (HI) across all sites was 0.55, a 20% increase compared with current cultivars. By tailoring phenology to the future weather patterns and selection of more drought-tolerant parameters, the effect of water stress on grain yield was reduced (Fig. 3E).

**Table 4.** Timing of anthesis and duration of grain filling (in days) of HT and HS ideotypes as simulated by Sirius for the HadGEM2-ES (RCP8.5) climate scenario at six European locations Information about sites is given in Table 2. Relative changes from current HT and HS cultivars are given in brackets.

Site	HT				HS			
	Mean anthesis		Grain filling duration		Mean anthesis		Grain filling duration	
TR	16 June	(+4.8)	61.7	(+13.3)	01 Jun	(–10.4)	61.9	(+13.5)
RR	26 May	(–9.4)	67.2	(+15.5)	26 May	(–9.7)	67.3	(+15.6)
DC	16 May	(+2.5)	54.7	(+10.1)	03 May	(–10.6)	51.4	(+6.8)
CF	18 May	(+6.3)	59.8	(+9.1)	04 May	(–7.7)	63.9	(+13.4)
MO	11 May	(+3.0)	56.2	(+9.9)	04 May	(–4.2)	58.0	(+11.7)
SL	11 Apr	(+0.7)	53.7	(+14.7)	12 Mar	(–29.2)	41.9	(+2.9)

As expected, grain yield of HS ideotypes were lower than HT ideotypes. The mean yield of HS ideotypes was slightly reduced at three sites, TR, RR, and MO, on average, by 3%. At the three other sites, CF, DC, and SL, the yield reduction was more pronounced, 7%, 16%, and 54%, respectively. In comparison with HT ideotypes, anthesis dates of HS ideotypes were brought earlier at all sites by 11.9 d, on average, compared with current cultivars (Fig. 3D). Maturity dates were similar to current cultivars at five of the sites but occurred 26.3 d earlier at SL. Consequently, the duration of grain filling of HS ideotypes compared with HT ideotypes was increased by 7% and 3% at CF and MO, similarly at TR and RR, and reduced by 6% and 22% at DC and SL. Despite relatively early flowering and grain filling, HSI95 remained high for DC and SL, 0.39 and 0.38, respectively (Fig. 3F). There is a trade-off between increasing yield potential with the higher risk of being affected by heat stress and achieving higher yield stability at the cost of lowering yield potential. For this reason, the mean grain yield of the HS ideotype selected at SL was 0.3 t ha^–1^ lower than the current HS cultivar, but its yield CV was only 0.14.

**Fig. 4.**
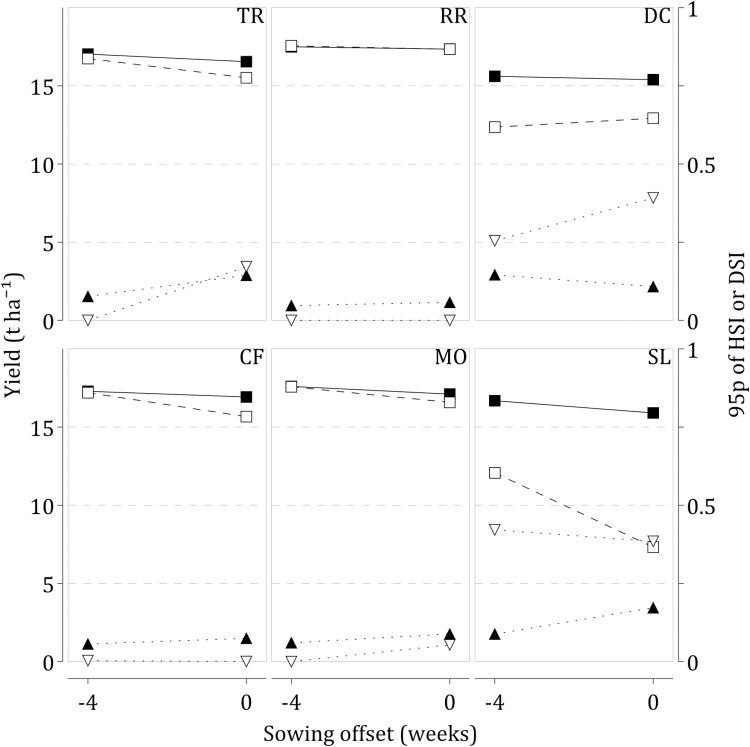
Mean grain yields for heat-tolerant (closed squares) and heat-sensitive (open squares) ideotypes, and 95-percentile of heat-stress index (HSI, open triangles) and drought-stress index (DSI, closed triangles) of heat-sensitive ideotypes for two sowing dates, default and 4 weeks earlier, as simulated by Sirius for the HadGEM2-ES (RCP8.5) climate scenario at six6 European sites. Information about sites and sowing dates is given in Table 2.

For these two sites, an earlier sowing allows for an extension of the growing season before high seasonal temperatures and either improves yields or reduces HSI95 (Fig. 4). At SL, the mean grain yield of the ideotype selected for the sowing date 4 weeks earlier was 12.7 t ha^–1^, a 65% increase relative to the current sowing date but HSI95 increased at the same time from 0.38 to 0.42. At DC, an earlier sowing did not improve mean grain yield but HSI95 was reduced from 0.39 to 0.25. The mean yield of HS ideotypes for the earlier sowing at these two sites is lower than the yield of HT ideotypes by 3.2 t ha^–1^ on average. An earlier sowing date provided limited adaptation to extreme temperatures and did not fill the yield gap between HS and HT ideotypes for these two sites, a result similar to Gouache *et al.* (2012). By contrast, at the other four sites, TR, RR, CF, and MO, an earlier sowing closed the yield gap between HS and HT ideotypes.

The impact of late frost was minimal for all ideotypes. The 5^th^ percentile of minimum canopy temperature +/–3 d around anthesis was always positive, on average 4.6 °C and 3.2 °C for HT and HS ideotypes, and the absolute minimum over the 100 years simulated reached negative temperatures only for the sowing date 4 weeks earlier. At CF, this absolute minimum was –0.3 °C for both HT and HS ideotypes. Absolute minimums of –0.5 °C and –0.4 °C were also recorded for HS ideotypes at DC and SL. No frost around anthesis was observed at the other sites. With the current implementation, the number of grains is reduced when the canopy temperature decreases below 0 °C and no grain are produced if it is below –1 °C. This narrow range of temperature strongly selects for ideotypes that delay anthesis until the probability of frost occurring around anthesis is very small. This is an additional constraint to limit early flowering of HS ideotypes. However, by contrast in Australian environments, for the European conditions explored here, there is a sufficient space between anthesis dates that minimize the risk of frost and anthesis dates that minimize the risk of heat stress (Zheng *et al.*, 2012).

In our simulations nitrogen (N) limitation was not considered. However, post-anthesis N uptake and redistribution could be a serious constraint in achieving high yield potential. In Sirius, grain demand for N during grain filling is satisfied from three sources (Jamieson and Semenov, 2000). The first is excess of N in the stem including N released by natural leaf senescence. If this amount is insufficient, then soil N is taken. The remaining demand for N is satisfied by remobilizing N from leaves and accelerating leaf senescence. As a result, grain-filling duration can be shortened and grain yield can be reduced. Increasing the capacity to store N in non-photosynthetic organs, such as internodes, which allows the translocation of N to grains without reducing wheat photosynthetic capacity, can prevent yield reduction (Dreccer *et al.*, 1997). Another strategy would be to improve post-anthesis N uptake from the soil. However, the ability of roots to take up N could decline during grain filling (Martre *et al.*, 2006). Moreover, in southern Europe, where grain filling coincides with low water availability, soil N available for uptake could be substantially reduced due to water shortage (Semenov *et al.*, 2007).

## Conclusions

Responses of wheat to high temperature during sensitive stages of wheat development, around anthesis and during grain filling, have been incorporated in the Sirius wheat model. The corresponding model parameters were calibrated using the Hot Serial Cereal experiment (Wall *et al.*, 2011; White *et al.*, 2011; Ottman *et al.*, 2012) and published data on a heat sensitive cultivar (Qin *et al.*, 2008; Vara Prasad and Djanaguiraman, 2014). This allowed us to assess yield losses for HS cultivars as a result of heat stress under future climate scenarios.

Wheat ideotypes were optimized for the HadGEM2-ES (RCP8.5) climate scenario at six European sites. Two types of ideotypes were investigated: fully tolerant to heat stress and heat-sensitive ideotypes based on parameters derived from the highly sensitive wheat cultivar ‘Chinese Spring’ (Qin *et al.*, 2008). This allowed us to quantify the uncertainty in assessing wheat yield potential as affected by future extreme weather events. An absolute tolerance to high temperatures is certainly unachievable. However, it has been shown that susceptibility to high temperatures during anthesis and grain filling varies among wheat cultivars (Alghabari *et al.*, 2014; Vara Prasad and Djanaguiraman, 2014). Therefore, impacts of heat stress on future yields could be expected between these two extreme cases. Our results demonstrated that a heat-tolerance trait is likely to become critical for southern and central Europe in the future in order to achieve high yield potential. With sufficient tolerance to heat stress, higher and more stable wheat yields could be developed by adapting the crop phenology to future weather patterns and extending the duration of grain filling. Wheat also benefited from maintaining leaf green area until the end of grain filling. In water-limited environments, particularly in southern Europe, drought tolerance, which delayed leaf senescence, was also a desirable trait. In SL, the optimal anthesis date for HS ideotypes was shifted to the beginning of March to avoid the impact of heat stress during flowering and grain filling, a month earlier compared with the HT ideotypes. This resulted in the substantial difference in grain yield for HT and HS ideotypes, with HT ideotypes achieving 15.9 t ha^–1^ yields and HS ideotypes achieving only half this yield (7.3 t ha^–1^).
